# Explaining Crossmodal Correspondences Between Colours and Tastes

**DOI:** 10.1177/20416695211018223

**Published:** 2021-06-20

**Authors:** Charles Spence, Carmel A. Levitan

**Affiliations:** Department of Experimental Psychology, Oxford University, UK; Department of Cognitive Science, Occidental College, Los Angeles, California, United States

**Keywords:** crossmodal correspondences, colour, taste, synaesthesia, emotional mediation, crossmodal statistics

## Abstract

For centuries, if not millennia, people have associated the basic tastes (e.g., sweet, bitter, salty, and sour) with specific colours. While the range of tastes may have changed, and the reasons for wanting to connect the senses in this rather surprising way have undoubtedly differed, there would nevertheless appear to be a surprisingly high degree of consistency regarding this crossmodal mapping among non-synaesthetes that merits further consideration. Traditionally, colour–taste correspondences have often been considered together with odour–colour and flavour–colour correspondences. However, the explanation for these various correspondences with the chemical senses may turn out to be qualitatively different, given the presence of identifiable source objects in the case of food aromas/flavours, but not necessarily in the case of basic tastes. While the internalization of the crossmodal statistics of the environment provides one appealing account for the existence of colour–taste correspondences, emotional mediation may also be relevant. Ultimately, while explaining colour–taste correspondences is of both theoretical and historical interest, the growing awareness of the robustness of colour–taste correspondences would currently seem to be of particular relevance to those working in the fields of design and multisensory experiential marketing.

The relation between colours and tastes has long fascinated mystics, artists, novelists, scientists, and latterly designers and marketers (e.g., Boehme, 1912, pp. 85–86; Classen, 1998; Heller, 1999; Spence, 2019a). However, the reason for their interest in this particular class of crossmodal correspondence has varied widely. While some commentators have chosen to focus on the synaesthetic colour concurrents that have been reported in rare cases of colour–taste synaesthesia (e.g., Kandinsky, 1977; Marks, 1978), others have been more interested in the potential that such crossmodal correspondences may hold in terms of helping to scale sensations across the senses (Tomasik-Krótki & Strojny, 2008). Other researchers, meanwhile, have used the existence of consistent crossmodal correspondences between colours and basic tastes to help establish the legitimacy of the latter (O’Mahony, 1983). For instance, the participants in O’Mahony’s study not only had to pick a colour to match each of the four basic tastes but also a day of the week, a state in the United States, and so forth. There was, in other words, nothing special about the colour–taste mapping in this study. Nevertheless, the idea that the four (or five) basic tastes, namely bitter, sweet, sour, salty, (and possibly also umami), can be meaningfully (or consensually) associated with particular colours (hues) is one that seemingly has widespread currency.

The linking of colours with basic tastes has surfaced in many different places over the centuries, from the fiction of Borges^1^ through to innovative marketing campaigns (such as orchestrated by Belgian paint company Boss back in 2015; Tastecolours, 2015). Recently, though, it has been the opportunities for multisensory design and experiential marketing that have emerged from connecting the senses in such surprising, almost synaesthetic, ways that have underpinned much of the commercial interest in this space (e.g., Dunne, 2014; Spence, 2012, 2020c; Spence & Youssef, 2019). But, one might ask, is there anything more to the idea of coloured tastes than an intriguing aesthetic conceit or a widespread fascination with the phenomenon of synaesthesia?

## A Brief History of Crossmodal Associations Between Colour and Taste

### Colour–Taste Synaesthesia

The suggestion that colours are connected to tastes (and flavours) has often reflexively been assumed to reflect a kind of synaesthesia. Indeed, over the past 130 years or so, there have been a number of (albeit occasional) reports of synaesthetes who have experienced colour concurrents in response to taste/flavour inducers. So, for example, Suarez de Mendoza’s (1890) early volume on coloured-hearing synaesthesia mentions one synaesthete who experienced a crossmodal association between a sour taste and the colour green (see also Schader, 1975, pp. 325–327). Meanwhile, in his influential tome, *The Unity of the Senses*, Lawrence Marks (1978, p. 95) refers to several case reports of synaesthetic coloured tastes, including reference to the work of Ginsberg (1923). The latter reports a synaesthete for whom sweet was associated with orange-red, salty with blue, sour with green, and bitter with black.

Here, it is interesting to note how many of the colour–taste matches that have been reported by the synaesthetes mentioned earlier would appear to match up fairly closely with the crossmodal correspondences between colour and taste that have been documented in non-synaesthetes (see Table 1 and the section “A brief history of crossmodal associations between colour and taste”). This is surprising inasmuch as synaesthesia is typically defined by the idiosyncratic nature of the inducer-concurrent relations that are experienced by those with the condition (e.g., Grossenbacher & Lovelace, 2001). Indeed, it is the regularity of the crossmodal mapping in the case of crossmodal correspondences that is part of what is thought to distinguish them from synaesthesia (see Deroy & Spence, 2013). That said, the existence of automatically-induced conscious concurrents in response to specific inducers also helps to differentiate the crossmodal associations that have been documented among non-synaesthetes from the peculiar experiences that are such a characteristic feature of synaesthesia proper.

**Table 1.** Summary of Results of Published Studies Showing Crossmodal Correspondences Between Colours and Basic Tastes.

Study	O’Mahony (1983)	Heller (1999)	Koch & Koch (2003)	Tomasik-Krótki & Strojny (2008)	Wan et al. (2014)	Woods & Spence (2016)	Woods et al. (2016)
Number of participants	51	2,000	45	519	452	201	200 (across two studies)
Origin of participants	USA (CA)	Germany	USA (OR)	17 countries	4 countries	MTurk (US?)	UK
Type of participants	University students	Cross-section of the public	Students	High school and university students	Internet recruits	Internet recruits	Internet recruits
Black	(Bitter)	Bitter	(Bitter)	–	Bitter	Bitter	Bitter
Blue		Salty		Salty		(Sweet/Salty)	Salty
Green	(Bitter)	Sour	Sour	Sour (Bitter)	Sour	Sour	Sour
Orange		Sweet	Sweet	Sweet			–
Pink	–	Sweet	–	–	Sweet	Sweet	Sweet
Red	Sweet	Sweet	Sweet	Sweet		(Sweet)	
Violet		Bitter		Bitter/Umami	–	Sweet	Sweet
White	Salty	Salty	Salty	–	Salty	Salty	Salty
Yellow	Sour	Sour	Sour	Sour		Sour	Sour
Brown		Bitter		–		–	–
Grey		Salty	–	–		–	–

*Note*. The participants in these studies had to pick one of the four or five basic tastes (words) to match a colour (word or colour patch), or else rate how well (or badly) the given taste matched a colour. The strongest crossmodal correspondences are shown, while weaker correspondences appear in brackets. “–” denotes that this colour was not tested in this study. Note also that not all of the colour options are shown for every study.

Importantly, however, the colour concurrents that have been reported by synaesthetes do not always appear intuitive to non-synaesthetes. So, for example, the synaesthetic artist Wassily Kandinsky mentioned a synaesthete for whom certain taste inducers give rise to blue colour concurrents. At one point, Kandinsky (1977) writes that *A Dresden doctor tells how one of his patients, whom he describes as ‘spiritually, unusually highly developed,’ invariably found that the certain sauce had a ‘blue’ taste, i.e., it affected him like the colour blue* (p. 158; see also Marks, 1978).^2^ More recently, Jaime Smith, a professional sommelier, describes how a particular white wine (Nosiola) has a *beautiful aquamarine, flowy, kind of wavy color to it* (quoted in Carlsen, 2013).

In conclusion, while a small number of cases of colour–taste synaesthesia have been reported in the literature over the past century of so, it is worth noting that coloured-taste synaesthesia appears to be a pretty rare form of the condition nowadays. According to Sean Day (2005), only 7.5% of the 572 synaesthetes quizzed reported experiencing coloured tastes. Thus, while the colour–taste mappings that have, on occasion, been reported by synaesthetes may sometimes bear an uncanny similarity to the ubiquitous colour–taste correspondences that have been reported by non-synaesthetes, synaesthesia would not appear to be the most appropriate explanation. Especially relevant factors to consider here are the consensuality of the mapping, not to mention the lack of any automatically-induced conscious colour concurrents linked to the basic tastes, in the case of non-synaesthetic colour–taste correspondences.

### A Brief History of Colour–Taste Correspondences

Interest in the putative links between colours and tastes goes way back in the historical record. For instance, Constance Classen (1998, p. 167) mentions how, in the 16th century, Hieronymus Cardanus classified Mars alongside blueness and saltiness, Venus was linked to whiteness and sweetness, while Saturn was linked to blackness and bitterness. Meanwhile, in the 17th-century, Gerónimo Cortés considered Mars to be red and spicy, Venus to be blue and musky, and Saturn to be black and acid.^3^ Moving forward to the opening decade of the 20th century, Japanese researcher Kikunae Ikeda (1909/2002, p. 849), when introducing the fifth taste “umami” to the world, suggested that “If these substances can be likened to color, ‘UMAMI’ would be yellow and sweetness red.” Meanwhile, according to Déribéré (1978), an applied researcher working in the field of fragrance marketing in France, the most popular colour associations for the four most commonly mentioned basic tastes are as follows: Salty: turquoise, light blue-green, white; Sweet: rose, white, red; Sour: lemon yellow yellow-green; and Bitter: brown-maroon, olive green.

In fact, over the past 50 years or so, a number of researchers have investigated the colour associations of basic tastes, or vice versa (see Tables 1 and 2 for a summary of the colour–taste associations that have been documented in various studies). As mentioned already, while the researchers’ motivation for assessing this specific crossmodal mapping has undoubtedly differed/changed, there is nevertheless still a surprising degree of consensuality in terms of the colours that people have chosen to match to each of the four basic tastes. Notice here also how the studies reported in Table 1 span a period of almost 40 years and incorporate the responses given by participants from many different countries, hence supporting the cross-cultural agreement regarding such seemingly “arbitrary” crossmodal mappings.

**Table 2.** Summary of Colour–Taste Recommendations For Designers (Favre & November, 1979; Spence et al., 2015) and Results of Published Studies (Déribéré, 1978; Saluja & Stevenson, 2018) Demonstrating Crossmodal Correspondences Between Taste Descriptors and Colour Words or Colour Patches.

Study	Taste	Colour correspondences
Déribéré (1978)		
	Sweet	Rose, white
	Sour	Lemon yellow, yellow green
	Salty	Turquoise, light blue-green
	Bitter	Brown-maroon, olive green
Favre & November (1979)		
	Sweet/Sweetish	Orange-yellow to red/pink
	Acid	Yellowish green to greenish yellow
	Salted	Grey, with pale green or with pale blue
	Bitter	Navy blue, brown, olive-green, violet
Spence et al. (2015)		
	Sweet	Red, pink
	Sour	Green, yellow
	Salty	White, blue
	Bitter	Black, purple
Saluja & Stevenson (2018)		
	Sweet	“Red and pink” (this is one category)
	Salty	Blue, “other colours”
	Sour	Yellow, “other colours”
	Bitter	Black, white, and grey; green
	Meaty	“Other colours,” red and pink

*Note.* The most popular corresponding colours, and combinations of colours, are listed first.

**Figure 1.**
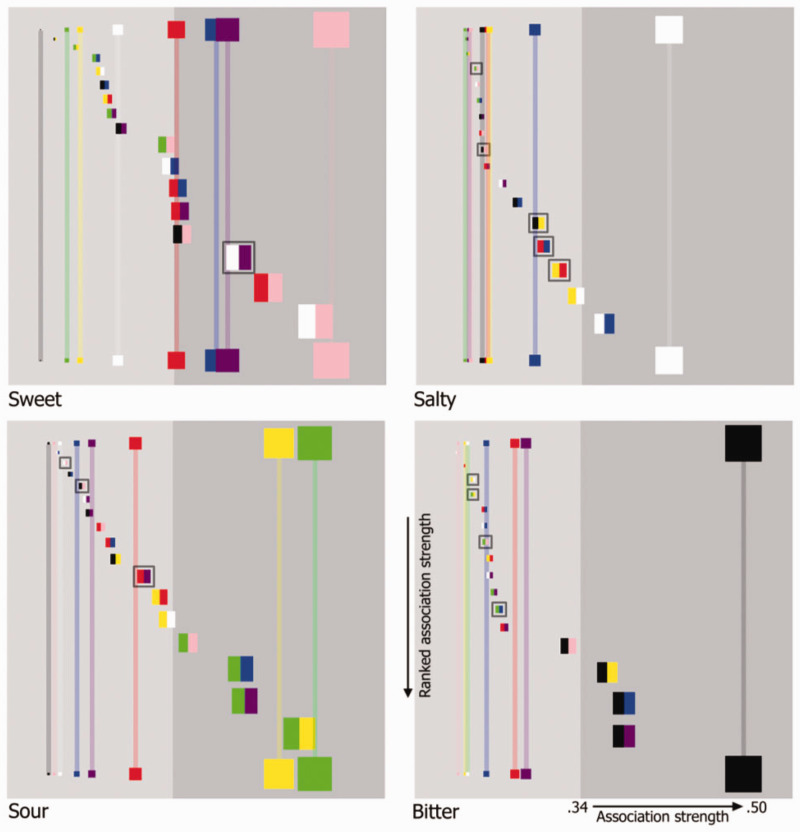
Four colour matrix plots depicting the frequency with which the different colours and colour pairs were selected for each of the four basic taste terms used in the study reported by Woods and Spence (2016). Now, the horizontal position of each patch represents the strength of association (as well as the size of each patch) with rightward-positioned patches being more strongly associated than those on the left-hand side (the more participants selecting a given colour for a given taste, the stronger the association; the horizontal axis being expressed as a ratio in terms of this value over the total number of participants). Single colour patches are presented horizontally along the top and bottom of each plot. In terms of their vertical placement, double colour patches have been arranged from the weakest association (top) to the strongest (bottom) in each plot. For illustrative purposes, double colour patches that were more strongly associated with a given taste than each of their constituent colours have been encapsulated in a grey highlighting box. The background of each plot was coloured light grey and dark grey, the latter signifying the region in which a particular taste was selected for patches by participants at a level that was significantly higher than expected by chance (*p* < .001). (Figure reprinted from Woods & Spence, 2016, Figure 7.)

More recently, Saluja and Stevenson (2018) assessed colour–taste correspondences using real tastants, in part to see whether the mapping would differ from those obtained when taste words are used instead. However, the results were essentially the same, thus suggesting that people have similar correspondences with taste words as with actual tastants. Furthermore, it is also intriguing to note that similar colour–taste correspondences are observed when participants associate one of the four basic tastes with abstract colour patches (see Figure 1; Woods & Spence, 2016).

Relevant here, Saluja and Stevenson (2018) recently tested 50 Australian participants with the five basic tastes delivered at three different stimulus intensities (cf. Wang et al., 2016). The participants were given a broad colour palate to choose from (they could also pick white, grey, or black; e.g., achromatic colours), and they were instructed to pick the colour that matched the taste that they were experiencing. The participants subsequently had to do the same in response to taste words rather than actual tastants. The results were fairly consistent no matter whether tastants or taste words were used. In both cases, the results were consistent with the majority of the previous research. While summarizing the research on such crossmodal correspondences between colours and tastes that had been published to date, Spence et al. (2015) concluded that pink and red were most strongly associated with sweetness, yellow and green with sour, white and blue with salty, and browny/black and purple (or possibly green) with bitter. The colours associated with the taste of umami have been less intensively investigated thus far and, what is more, haven’t yet led to especially consistent results (see Tomasik-Krótki & Strojny, 2008; Wan et al., 2014 for the only two studies to have assessed the colour associated with umami; though note that Saluja & Stevenson, 2018 assessed the colours people associated with “meaty”).

### How Many Basic Tastes Are There, and Do They All Have Colours?

While the majority of the research on crossmodal correspondences between colour and taste that has been published to date has tended to focus on the four so-called basic tastes (sweet, sour, bitter, and salty) that most of us are familiar with in the West, there has, as we have just seen, occasionally also been suggestions regarding the fifth taste, that of umami (Ikeda, 1909/2002). Beyond that, it is worth noting (especially given the historical bent of this review) how other taste categories were often mentioned at certain points in history. For example, according to Chris Woolgar, in Late Medieval England, at least eight different tastes (along with tasteless) would have been recognized (Woolgar, 2006). They comprised sweet, greasy, bitter, salty, sharp, harsh or styptic,^4^ salty like the sea, and vinegary. One might legitimately ask whether these less familiar tastes were also once associated with colours. We are, however, unaware of any evidence on this particular score.

And, looking to the present/future, various other “basic” tastes have been put forward by researchers (thus far with varying degrees of support). These include metallic (e.g., Reith & Spence, 2020; though see also Ikeda, 1909/2002), starchy (Lapis et al., 2016; though, once again, see also Ikeda, 1909/2002), calcium (Tordoff et al., 2012), and fatty (acid) taste (e.g., see Mattes, 2009; see also Chale-Rush et al., 2007; DiPatrizio, 2014). One might wonder whether these putative basic tastes (though see Delwiche, 1996; Erikson, 2008; Hartley et al., 2019) also have colours, or other visual appearance properties, associated with them. Do people, for example, tend to associate a metallic taste with a metallic appearance, as the linguistic/semantic account of crossmodal correspondences might be taken to suggest (see Gallace & Spence, 2006; Martino & Marks, 1999; Spence, 2011; Walker & Smith, 1984)? This would seem unlikely given that a metallic taste is presumably much more likely to be experienced in the context of blood. As such, the crossmodal association might rather be with a dark red colour instead (i.e., if the source object that comes to mind on experiencing a metallic taste is blood).^5^ That said, we are not aware of any data having yet been collected on the colour associations of metallic taste sensations. This is despite the growing interest in the latter, given their increasingly frequent occurrence among those undergoing chemotherapy (e.g., Pirkwieser et al., 2021; Reith & Spence, 2020).

One of the limitations with the colour–taste correspondences research that has been conducted to date is that it has tended to focus only on the four (or, on occasion, five) basic tastes that people are most familiar with nowadays and to have neglected the various other tastes for which there is currently varying degrees of empirical support (see Anonymous, 2015). To give some sense of what might be missing from a comprehensive mapping of colours to tastes, in her popular science book *Taste What You’re Missing: The Passionate Eater’s Guide to Why Good Food Tastes Good,* Barb Stuckey reports how a number of the food scientists she interviewed believe that there are as many as 20 or more basic tastes (Stuckey, 2012)! At the same time, however, it is also worth noting that the colour correspondences for taste that have been reported in the literature have sometimes been constrained by the limited range of colour terms that the participants have been given to choose from. So, for example, while people typically pick pink as a good match for sweetness when that response option is available to them, it has not always been available. This methodological limitation can then potentially help to explain the differing colour matches that have sometimes been reported in different studies of colour–taste correspondences (see Spence et al., 2015).

### Interim Summary

The research that has been summarized in this section clearly demonstrates that people have been minded to connect colours to tastes in a seemingly consistent (i.e., consensual—or commonly agreed) manner for centuries. However, having demonstrated the cross-cultural and trans-historical robustness of these crossmodal correspondences between colour and taste (at least for the four or five currently most commonly accepted basic taste categories), we can now move on to consider what the most appropriate explanation for such surprising correspondences might be. Over the years, a number of different suggestions have been put forward in the literature to account for the general class of crossmodal correspondences (see Spence, 2011; Spence & Sathian, 2020 for reviews). Specifically relevant to the case of colour–taste crossmodal correspondences, these include explanations framed in terms of crossmodal similarity, statistical learning, and perhaps also emotional mediation. In the next section, the explanatory value of each of these accounts will be assessed.

## Explaining Colour–Taste Correspondences

### Are Colour–Flavour Correspondences Based on Crossmodal Similarity?

One might wonder whether crossmodal correspondences between colours and tastes might somehow be based on the perceived crossmodal similarity of the component unisensory stimuli. However, as Hermann Helmholtz, the eminent early German psychophysicist, argued long ago:

The distinctions among sensations which belong to different modalities, such as the differences among blue, warm, sweet, and high-pitched, are so fundamental as to exclude any possible transition from one modality to another and any relationship of greater or less similarity. For example, one cannot ask whether sweet is more like red or more like blue. Comparisons are possible only within each modality; we can cross over from blue through violet and carmine to scarlet, for example, and we can say that yellow is more like orange than like blue! (Helmholtz, 1878/1971, p. 77)

At this point, it is perhaps relevant to note that Helmholtz does not distinguish between the senses of taste and smell, especially given that olfactory stimuli (in contrast to visual stimuli) have been documented to take on the perceptual qualities of the tastes that they commonly co-occur with in food (e.g., Blank & Mattes, 1990; Stevenson & Boakes, 2004). Consider here only how most of us perceive vanilla to smell sweet, despite the fact that vanilla pods actually taste very bitter. The acquisition of taste properties by odorants means that food aromas can, over time, sometimes end up becoming more similar to the tastes with which they are commonly associated (Jones et al., 1978). As such, people are able to make meaningful judgements of the perceived similarity of pure gustatory and olfactory stimuli (e.g., as when judging the similarity of sugar to various aromatic spices in an intriguing study by Blank and Mattes, 1990), thus seemingly contradicting the general claim made by Helmholtz. Importantly, however, visual stimuli do not appear to take on, or acquire, the perceptual qualities of the tastes they are correlated with. This, then, is one of the fundamental differences between vision (colour) and olfaction (see also Baeyens et al., 1990; Christensen, 1983, p. 790).

Sometimes, though, parallels are drawn between the senses in order to highlight the similar principles operating within the different senses rather than necessarily to assert the crossmodal similarity of the component unimodal stimuli. For example, in *The Art of Perfumery,* first published in 1855, the Paris-based English chemist and perfumer Septimus G. W. Piesse famously drew a parallel between olfactory stimuli and musical notes with his scent scale (see Deroy et al., 2013).^6^ However, a closer reading of Piesse’s seminal work suggests that his interest was primarily on bringing out the similarity of the intramodal relations that are observable within each sensory modality rather than necessarily the crossmodal similarity between specific olfactory stimuli and auditory notes.

Taken together, while it might make sense to rate the similarity of olfactory and gustatory stimuli (as the former can take on properties of the latter following pairing), there is little evidence to suggest that people experience perceptual similarity between colours and basic tastes (see also Tversky, 1977 on the topic of similarity). That said, it is worth noting that perceptual similarity, in terms of intensity matching, might well influence the saturation of colour given for tastes having differing intensities. Relevant to this point, Saluja and Stevenson (2018) reported that colour saturation was related to tastant concentration. Thus, while basic taste-hue mappings appear not to be driven by crossmodal similarity, the *intensity* of the hue may be (e.g., light pink for less sweet tastes/foods, and fuchsia/hot pink for very sweet tastes/foods). 

Food colours undoubtedly do help to set our taste expectations (Piqueras-Fiszman & Spence, 2015b), given that colours are correlated with taste properties (as we will see in the next section). Here, it is interesting to note that people have more often chosen to pair the basic tastes with colours than with auditory (or even tactile) stimuli. The popularity of this particular pairing might thus be taken to suggest that there is some more fundamental affinity between basic tastes and colours (more so than, say, between basic tastes and musical notes). By contrast, consider only how olfactory stimuli (and fragrances) are often connected to the language of music—everything from high and low notes, through chords and harmonies (language terms that can be used to describe both auditory and olfactory stimuli; see Deroy et al., 2013), and not forgetting Piesse’s (1862/1891) early suggestions on this score (namely his scent scale).

### Internalizing the Crossmodal Statistics of the Environment

Given that neither colour–taste synaesthesia nor crossmodal perceptual similarity appears capable of providing a satisfactory explanation for the long-standing, and largely consensual, crossmodal associations (or affinities) that so many of us seem to feel between colours and basic tastes, what other explanation might there be for this ubiquitous phenomenon? While people are undoubtedly capable of rapidly learning associations between colours and tastes (Higgins & Hayes, 2019), it is important to stress that the senses of gustation and vision actually pick up on very different kinds of sensory information.^7^ Relevant here, crossmodal correlations are likely to be strongest in those cases where the different senses pick up on the same “amodal” stimulus property (e.g., Lewkowicz & Turkewitz, 1980; though see Spence et al., 2013). Given that colour and gustation pick up on very different environmental properties, the statistical correlation is likely going to be somewhat weaker than in the case where, say, both vision and touch provide information about the size or shape of an object (Ernst & Banks, 2002).

Perhaps the most parsimonious explanation for the ubiquity of colour–taste correspondences is in terms of the internalization of the crossmodal statistics of the environment (e.g., Barlow, 2001; Ernst, 2007). There is, for example, evidence from both adults (Higgins & Hayes, 2019) and also 4-month-old babies (Reardon & Bushnell, 1988) that, regardless of their age, people rapidly pick up novel associations between colours and basic tastes. It would certainly seem unlikely that we have developed hardwired associations between specific colours and particular tastes, given the changing association between taste/flavour and colour both across cultures, and over the course of history (e.g., Hisano, 2019; Woolgar, 2018; see also Wilson, 1991).^8^ Here, one might only consider how blue colouring in foods came to be widely associated with sweet-tasting flavours—be it blue raspberry (in candy floss or soft drinks) or blue curacao; Spence, 2021a). Recently, Woolgar highlighted how certain dishes were associated with widely different colours during the Middle Ages. Intriguingly, Woolgar describes a number of savoury blue dishes (potentially coloured using a lichen with the right level of acidity/alkali) from the Middle Ages including a minced meat dish with the most unappealing name “blue mush”! Hence, given that the sweet-blue association presumably did not exist (in Europe) prior to the 20th century, but is robustly represented in the marketplace today, this once again underscores the fact that such correspondences are likely learned, at least in part, via experience.

It is perhaps worth distinguishing here between natural and artificial crossmodal (cor-)relations (in the language of Walker-Andrews, 1994). Correlations between redness and ripeness in fruits (Foroni et al., 2016; Lee et al., 2013), for instance, as well as between blue/purple colours and phytonutrient content in various fruits and vegetables (Pinto & Vilela, 2021), and possibly also between green produce and bitterness, are all examples of natural (arbitrary) crossmodal relations. Importantly, however, the rise of synthetic chemistry rapidly/suddenly increased the possibility of artificially colouring foods around the turn of the 20th century (Blaszczyk & Spiekermann, 2017; Hisano, 2019).^9^ Many of the crossmodal colour–taste associations that consumers are exposed to nowadays are thus examples of what Walker-Andrews (1994) refers to as “arbitrary/artificial” (think here only of blue coloured-raspberry flavoured drinks; Shankar et al., 2010; Spence, 2018b, 2021a).

The basic suggestion is therefore that the vast majority of colour–taste correspondences in the (non-synaesthetic) general population are unlikely to reflect innate connections between the senses but are rather presumably acquired based on the internalization of the crossmodal statistics of the environment. While certain of these correlations between colour and taste can be described as natural (albeit arbitrary), others are artificial (and arbitrary; according to Walker-Andrews’, 1994, categorization of crossmodal relations). However, the claim that the ubiquitous existence of consensual crossmodal correspondences between colours and basic tastes can largely be explained in terms of the internalization of the correlations between colour and taste that we experience still leaves open a number of important questions that need to be addressed in order to flesh out the predictions/constraints that such an account would entail. It is to these questions that we turn next.

#### Is the Crossmodal Mapping Between Colour and Taste Mediated by a Specific Source Object?

One key question that has not as yet been addressed in the literature on colour–taste correspondences is whether they are always mediated by a particular source object. So, for example, when people report that a transparent blue drink looks sweet, one might ask whether the association between colour and taste is direct, or whether instead it is mediated by reference to a particular flavourful food. In this case, the likely source object might well be one of those popular blue raspberry-flavoured drinks that are available in the marketplace nowadays (see Spence, 2018b, 2021a; Spence & Levitan, 2021 on this theme).^10^

Discussion of coloured tastes often occurs in the context of coloured smells and coloured flavours (e.g., Déribéré, 1978; Piqueras-Fiszman & Spence, 2015a). Intriguingly, a number of the researchers working on crossmodal correspondences between colours and odours/fragrances have demonstrated that the colours that people pick for odours are often determined by the identification of a particular source object for the odour (that is true regardless of whether they bring to mind the correct source object or not; see Spence, 2020c for a review). That is, odour/flavour correspondences with colour are often based on semantic knowledge or specific exemplars—oranges smell of orange and are orange-coloured, bananas are typically yellow and smell of bananas (though, as Hisano, 2019, has pointed out, a century ago, the skin of oranges was as likely to be green as orange, while red-skinned bananas were almost as common as the yellow-skinned varieties that are so popular today). One potentially important difference is that basic tastes are properties of many/all flavourful stimuli but largely do not constitute source objects in their own right. Of course, nowadays everyone is presumably familiar with sugar and salt as providing access to the relatively pure basic tastes (though, at the same time, it is worth noting that the ingredients [and thus tastes] are rarely consumed in isolation but are instead typically added to other foods/drinks).^11^ Hence, it is natural to assume that nowadays white salt and refined white sugar crystals might well constitute the source object that springs to mind when people are instructed to pick the corresponding colour for a salty or sweet taste.^12^

It is important to stress that while there are undoubtedly some relevant similarities between different types of crossmodal correspondence involving colour and the chemical senses (specifically, gustation, olfaction, and flavour),^13^ there are likely also a number of potentially important differences as well (e.g., Levitan et al., 2014; see Spence, 2020c for a review). For instance, consider only how basic tastes (such as sweet and salty) are typically thought of as features (or attributes) of foods (perhaps in the same way as meaty, floral, herbal, burnt aromas/flavours) rather than as source objects/semantically meaningful stimuli in their own right. What is more, people typically struggle to identify the source object when olfactory stimuli are presented in the absence of any other identifying information. The dimensionality of odour is, of course, also very different to that of taste; humans have ∼1,000 different receptor types for odours and yet struggle to identify odours (Yeshurun & Sobel, 2010; though see Majid & Burenhult, 2014, for a striking counterexample of the Jahai, who can name odours and colours equally well). Schloss et al. (2015a) found that participants collectively named an average of 53 objects for each odour in their study (exceeding the number of participants, 32, who were tested), thus demonstrating that people tend to generate multiple possible sources objects when presented with an odour. By contrast, with the four main basic tastes, there is far less ambiguity, excepting the occasional confusion between sour and bitter (Doty et al., 2017; Hettinger et al., 1999; O’Mahony et al., 1979). Of course, as soon as one goes beyond the basic four, many of us soon start to struggle.

#### Multiple Colours Correlated With the Basic Tastes of Food and Drink

If you are asked to come up with sweet-tasting foods, you will likely mention a wide range of products (that coincidentally have a variety of different colours). Your list might, for example, include foods such as cola (dark caramel browny-black in colour), ice cream (white, pink, brown, light green, etc.), sugar (white or brown), honey (golden brown), or candy (a broad range of colours in terms of both the candy itself as well as its packaging). The problem, in other words, when it comes to colour–taste correspondences is unlikely to be a difficulty in identifying relevant source objects (as is the case for colour-odour correspondences) but rather the fact that there are numerous different relevant source objects (i.e., foods) to choose from. Hence, given that various different colours are associated with foods having each of the basic tastes,^14^ the question immediately arises as to how this ambiguity is resolved when people spontaneously pick a corresponding colour for one of the basic tastes.

One possibility here is that people simply go with the colour of the first food that happens to come to mind when picking a colour to match a given basic taste.^15^ However, research on people’s preferences for colours and for odours suggests that some kind of averaging model that includes multiple associations works better than one based on just a single exemplar (see Palmer & Schloss, 2010; Schloss et al., 2015a). Furthermore, when people’s own idiosyncratic object associations (e.g., the colour red might remind someone of their favourite pair of red trousers) are included, as well as “standard” associations generated across a group (e.g., the colour blue for the sky), the fit of such preference models improves still further (Schloss et al., 2015b; Schloss & Palmer, 2017).

Given such findings, Spence and Levitan (2021) recently considered whether that colour–taste crossmodal correspondences might also be based on some sort of synthesis of crossmodal associations rather than merely on the top-of-mind association of a specific stimulus having the desired taste. That is, a basic taste quality (either physically experienced or merely imagined) might well activate expectations concerning several different colours in parallel, perhaps with different strengths (or weights), depending on an individual’s particular experiences, as well as the context (Elliot & Maier, 2012). Here though, it is worth highlighting how such an averaging process would appear to be quite different from the aligning of stimulus dimensions that has been studied by the likes of Ernst (2007), when investigating the acquisition of novel crossmodal correspondences. In the latter study, for example, participants learned to correlate two unisensory signals that each varied along only a single dimension (see Spence, 2011).

A fundamental problem that arises as soon as one tries to apply a weighted averaging account to taste-colour correspondences is that while it might work for continuous prothetic dimensions (such as rated pleasantness in studies of Ecological Valence Theory), where all values can be placed along a single more-than/less-than dimension, such averaging does not so obviously make sense for metathetic categorical perceptual dimensions such as colour (hue) category and the categories of basic taste (see Spence, 2011, Stevens, 1957 on the distinction between metathetic and prothetic dimensions).^16^

Another issue to consider here relates to the various memory biases that may come into play whenever people rely on the availability heuristic (e.g., Pollard, 1982; Tversky & Kahneman, 1973), such as the overweighting of recent or particularly salient information. In the typical study of source objects in the context of colour–taste mappings, the participants have typically been asked to justify their colour-choice after having made a colour choice in response to a taste (or taste word), for example, “I choose yellow for sour, because of lemons.” What this means, in practice, is that is not altogether clear whether they are confabulating feasible explanations for their colour-choices—or whether the source object really did come to mind when experiencing/imagining that basic taste. Such a memory/post hoc bias could potentially be controlled for simply by concealing the study aims—for instance, by getting participants to taste a sweet, salty, sour, bitter, and umami solution, and asking them to describe the objects that come to mind (in terms of their shape, size, colour, use, familiarity, and so forth; cf. Ngo et al., 2013).

In conclusion, it would seem likely that the various consensual crossmodal correspondences that have been documented between colour and taste are mediated by source objects. However, in contrast, to the case of colour-odour correspondences (where people often struggle to bring to mind the appropriate source object for a scent when it is presented without any contextual information), in the case of colour–taste correspondences, the problem is likely to be one of how to decide between the many differently coloured foods that people tend to associate with each of the basic tastes (given that basic taste qualities constitute typical properties of a wide variety of foods, rather than source objects in their own right, as is often the case for odours; Hutchison, 2003).

### Emotional Mediation of Crossmodal Correspondences

Before concluding this section, it is worth considering another increasingly popular explanation for many crossmodal correspondences in terms of emotional mediation (sometimes referred to as affective correspondences; Palmer et al., 2013; Schifferstein & Tanudjaja, 2004; Whiteford et al., 2018). Emotional mediation provides an alternative explanation for those crossmodal correspondences where it is difficult to formulate a statistical account (i.e., where there is no obvious source object possessing the corresponding sensory properties; Wang et al. 2016, and see Spence, 2020a for a review). According to the emotional-mediation account, the reason why, for example, sweetness and the colour pink are so often associated might be because both stimuli are independently linked with happiness. By contrast, bitterness and blackness might both be associated with negative emotions. Relevant here, emotional mediation is also one of the dominant explanations for the crossmodal correspondences that have been documented between basic tastes and shapes (e.g., Velasco et al., 2016). According to an alternative account, the perceived spatiotemporal similarity in sensations (Obrist et al., 2014) may provide an alternative basis for the mapping. However, regardless of which explanation one prefers, the correspondence is not based on the shape of the foods in which those taste properties happen to be present (see Spence & Deroy, 2012, 2013).^17^ Consider here how only if we extended the suggestion that the white-salty association is based on salt crystals, sugar crystals are also white and highly angular. Yet people typically match sweet tastes with round shapes not angular ones.

Intriguingly, a number of researchers have suggested that emotional mediation can be used to explain certain colour-odour correspondences, especially in the case of fine fragrances (e.g., Kim, 2008, 2013; Schifferstein & Tanudjaja, 2004). In such cases, no obvious source object is likely to come to mind, contrasting with what normally happens when people are presented with the scent of a fruit, herb, or spice, say (see Spence, 2020d for a review). Colour correspondences to odours that are unlikely to have been experienced previously have, on occasion, also been documented (e.g., see Spence, 2020c), again suggesting that there need not be an identifiable source object.

One open question, though, for the emotional-mediation account of crossmodal correspondences is which emotions to consider and whether they should be presented as bipolar dimensions or not. For example, Whiteford et al. (2018) used 10 separate scales (happy/sad, calm/agitated, complex/simple, appealing/disgusting, loud/quiet, spicy/bland, warm/cool, whimsical/serious, harmonious/dissonant, and like/dislike), some of which are not obviously reflective of emotions, per se. By contrast, Schifferstein and Tanudjaja (2004) used 15 items that they subsequently collapsed onto three dimensions (pleasure, arousal, and dominance), while Wang et al. (2016) used the two popular dimensions of valence and arousal.

In contrast to the case for olfaction, it may be that the emotional space is much simpler for taste (gustatory) stimuli. According to one hypothesis, the function of basic tastes is to provide us with information about whether to consume (i.e., approach) or avoid (e.g., sweet and fatty foods are almost universally liked, whereas bitter foods tend to be liked less, which could be evolutionarily advantageous, given that many poisonous foods have a bitter taste (see Drewnowski, 1997; Piqueras-Fiszman et al., 2014). According to the latter view, one might imagine liking and dislike/disgust to be the most appropriate anchors. Another intriguing direction to pursue here might be in terms of the semantic differential technique (e.g., Osgood et al., 1957; Snider & Osgood, 1969). However, while this approach has been used to categorize the meaning (or association) of both colours (Adams & Osgood, 1973) and odours (Dalton et al., 2008), we are not aware of anyone yet having conducted a semantic differential analysis of the basic tastes.

Here, it should also be remembered that depending on culture, colour can have a very specific meaning in the context of food. In Japanese cuisine, for example, there is a practice of balancing five colours: red, blue/green, yellow, black, and white. Interestingly, the different colours serve different roles: Red and yellow are associated with warming up the appetite; blue/green is meant to evoke refreshment; white evokes notions of cleanliness; and black provides contrast (Anonymous, 2020). But, one might ask, are these meanings associated with particular tastes, or is it the combination of colours that creates a feeling of harmony and balance? Outside the world of food, there is an extensive marketing literature concerning the sometimes striking differences in the emotional connotations that different colours have in different countries/cultures (e.g., Labrecque et al., 2013; Spence, 2021b; Wheatley, 1973).

One further issue that it is important to bear in mind for the affective account of crossmodal correspondences involving colour is that people’s colour preferences change markedly depending on whether they are considering colour in the abstract versus in the context of food. Consider here only how blue is typically chosen as the favourite colour (e.g., Hurlbert & Ling, 2007; Palmer & Schloss, 2010; though see Taylor et al., 2013 for a cross-cultural counterexample). By contrast, in a food context, blue is much more likely to appear at the bottom of the list of preferred colours, being associated with unnatural foods (see Spence, 2019b), whereas red foods tend to be more liked (e.g., Lee et al., 2013). At the very least, then, the emotional-mediation account of colour–taste correspondences needs to be sensitive to the context-dependent affective associations that different colours may evoke/prime (Elliot & Maier, 2012; cf. Schietecat et al., 2018).^18^

### Interim Summary

While a number of distinct explanations/accounts have been put forward over the years in order to try and explain the consensual crossmodal correspondence between colours and basic tastes, the internalization of the statistical regularities of the environment would appear to provide the most parsimonious account for the majority of the data that has been collected to date. Alternative suggestions in terms of (synaesthesia and) crossmodal perceptual similarity appear less plausible. The emotional-mediation account of crossmodal correspondences has become increasingly popular as an explanation for those crossmodal correspondences where a statistical explanation appears implausible, as when particular colours are chosen to correspond to specific pieces of music (Palmer et al., 2013) or paintings (see Spence, 2020a for a review).

The emotional-mediation account has also been used to help explain the crossmodal correspondences that have been documented between unfamiliar fragrances and colour patches (e.g., Kim, 2008, 2013; Schifferstein & Tanudjaja, 2004; see Spence, 2020d for a review), as well as the crossmodal correspondence between shapes and tastes (see Velasco et al., 2016). As such, it would seem sensible not to ignore the potential emotional mediation of colour–taste correspondences too, especially when the correspondence is between abstract stimuli—such as colour patches and basic taste words (i.e., where there is no obvious link to a source object). That being said, one potentially interesting question for the future is to consider whether people’s colour choices for basic tastes are based either on source objects/statistical regularities or on emotional mediation, or whether instead some hybrid colour choice can be operationalized that combines inputs from both the statistical and emotional-mediation accounts.

It should be stressed that the colour choices that people make in response to basic tastants and taste words may ultimately reflect some combination of the different explanations that have been put forward here. Perhaps the weighting of the different accounts might actually differ as a function of the specific taste quality under consideration. Perhaps the robustness of the sweet-red, green-sour association is linked to the ripening of fruits and/or the ubiquity of lemons/limes as a source object that rapidly comes to mind. By contrast, the responses to unpleasant bitter tastes may weight more heavily on emotional mediation instead, with people picking colours that they don’t like to match a taste that they don’t like. Appetitive and non-appetitive tastants might also dissociate. Furthermore, one other question for future research may be to determine what colours are chosen for combinations of basis tastes (acknowledging the ubiquitous mutual interactions that have been documented to occur between the basic tastes) or pairs of taste words, such as, for example, “bittersweet” (which may bring to mind grapefruit as the source object) or “sweet and sour.”

## Incorporating Colour–Taste Correspondences Into “Synaesthetic” Design and Experiential Marketing

**Figure 2.**
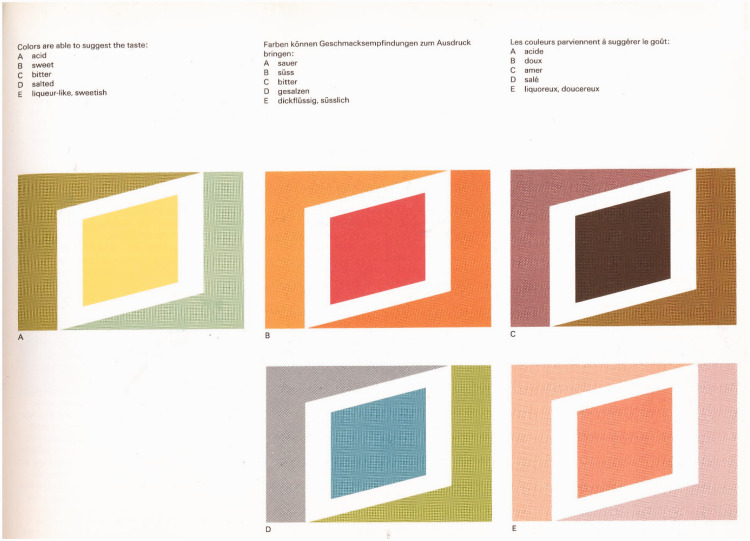
Abstract patterns designed to convey distinct tastes. Does it make sense to consider the correspondence between such visual designs and basic tastes in the same way as when considering unique colour features? (Reprinted from Favre & November, 1979.)

As soon as one moves from the literature on crossmodal correspondences into the world of design, people start to combine multiple colours to in order to convey information about the taste of a product while also, increasingly, manipulating the curvilinearity/angularity of shapes and background elements as well. Nevertheless, the evidence suggests that the more cues (e.g., both colours and shapes) that are combined, the less ambiguous the intended meaning (or correspondence) is. According to research from Woods and his colleagues, for example, pairs of colours, especially when arranged as foreground/background colours can be more strongly associated with a particular taste than the best of the individual colours (e.g., Woods et al., 2016; Woods & Spence, 2016). Perhaps unsurprisingly, there has been growing interest from the world of marketing and packaging design, in how best to convey the sensory attributes of food products by means of the abstract use of colour and shape/form (e.g., Fateminia et al., 2018, 2020; Favre & November, 1979; Lick et al., 2017; Matthews et al., 2019; Sugrue & Dando, 2018; and see Spence, 2020d for a review). Intriguingly, in their 1979 book on colour marketing, Favre and November present graphical representations to convey the basic tastes (see Figure 2). Although the shapes/form may seem stylized in this case, the use of combinations of colours can be seen as way ahead of its time (see Spence, 2020d for a review).

Once colour–taste correspondences are extended beyond coloured food and beverage products, then that suddenly opens up a world of possibilities in the field of design. Indeed, research conducted over the past decade or so clearly shows that people readily associate basic taste properties with colours even when they are evidently not the colour of something edible. So, for example, over the past decade or so, it has been shown that the colour of (coffee) cups (Carvalho & Spence, 2019), the colour of ambient lighting (Spence et al., 2014), and even the dominant hue of natural environments (Motoki, Takahashi et al., 2021; see also Chen et al., 2020) are all readily associated with, and can very often influence, taste perception. Unsurprisingly, the graphic design (e.g., incorporating colour, form and typeface) of product packaging has also been shown to influence taste perception in many, if not all, cases (see Fateminia et al., 2018, 2020; McDonald, 2018; Sugrue & Dando, 2018; Velasco & Spence, 2019; Zellner et al., 2018).

Many marketers have taken note of colour–taste correspondences as a way to energize their advertising campaigns. For instance, Boss, a Belgian paint company, worked together with a group of top chefs, including chocolatier Dominique Persoone, to match their range of paint colours to basic tastes (see Tastecolours, 2015). The colour–taste mappings match up pretty closely with Heller’s (1999) suggestions (see Table 1). Meanwhile, Pantone declared Classic Blue (19-4052) its colour of the year in 2020. According to Fixsen (2019),

To augment the 2020 reveal, Pantone included a twist of its own: As part of its marketing campaign, the company partnered with several brands to develop the smell, sound, taste, and texture of Classic Blue. The resulting package included a swatch of suede-like fabric from the Inside, a musk-and-sea-salt-scented candle, a blue, berry-flavored jelly, and a three-minute audio track titled “Vivid Nostalgia.”^19^

This campaign seems almost synaesthetic, suggesting as it does that the particular shade of blue can be meaningfully associated with a specific taste/flavour.

In another project, one of the authors (C. S.) worked together with Guinness and creative agency RGA+, to create a series of colourful audiovisual virtual reality animations designed to bring out the taste/aroma/flavour attributes of three drinks (Glenday, 2017; Hills-Duty, 2017). Meanwhile, working with U.S. pop group, The Roots, C. S. also fed into the creative process leading to a video that was developed for a song called “Sweet till the bitter end.” The colours and shapes in the background of the music video, along with the instrumentation, were designed to bring out either the sweeter or more bitter notes in the beer (e.g., Reed, 2016). Others, meanwhile, have combined shape and colour to bring out sourness/acidity (e.g., Huisman et al., 2016; see also Wu & Gingrich, 2020).^20^

**Figure 3.**
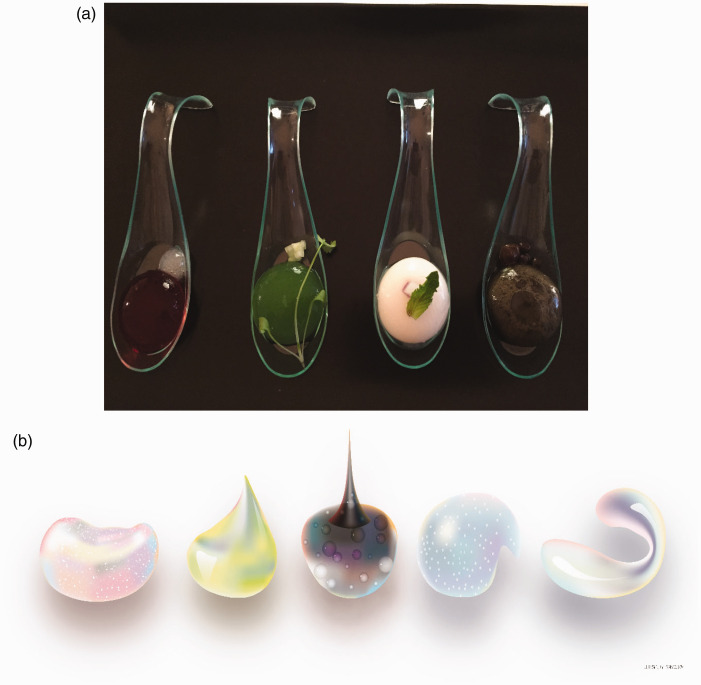
(A) Salty, bitter, sour, and sweet, the *amuse bouches* served at Synaesthesia by Kitchen Theory (see https://kitchen-theory.com/). The spoons were brought to the table in a random arrangement. The diners were instructed to sort the tastes by means of colour. The spoons in the figure are arranged in the order (sweet, sour, salty, and bitter, from left to right). This dish was inspired by the latest cross-cultural research demonstrating the robust crossmodal correspondences that exist between colour and taste. (B) The five basic tastes (sweet, sour bitter, salty, and umami) as conceptualized by London-based designer Jialin Deng for a range of tableware. (http://jialindeng.wix.com/whispery-savoury) (Figures reprinted from Spence et al., 2015.)

As an example of the kind of synaesthetic campaign that has become especially popular in recent years, Schwartz used exploding bags of colour(-ed spices) in an advertising campaign for their Flavour Pods range (see Dunne, 2014). Meanwhile, Chef Jozef Youssef of Kitchen Theory in London has been serving an amuse bouche as part of his gastrophysics chef’s table and synaesthesia menus in which four spherified coloured balls of taste were presented to the diner (see Figure 3A). These were placed in a random order in front of the diner who was then encouraged to rearrange their spoons from left to right in the tasting order; salty, bitter, sour, and sweet (Spence et al., 2015; Spence & Youssef, 2019). At around the same time, designer Jialing Deng created a range of prototype plates designed to match each of the five basic tastes (see Figure 3B). The majority of people were able to decode the basic taste associated with each of the plate designs.

What is striking about the examples discussed in this section is how synaesthesia still provides an attractive starting point for many in the creative industries. What the examples highlighted in this section also make clear is how in any real-world situation, there are always a host of contextual cues, and likely multiple sensory features, that presumably help to constrain the crossmodal mapping (cf. Spence, 2020b). This contrasts with the majority of the laboratory studies reviewed in the Explaining colour–taste correspondences section, where only a single taste and colour were to be paired, often in the absence of any contextual information whatsoever.

## Conclusions

As this review of the literature has clearly demonstrated, there is a long history of people wanting to connect colour (hue) categories to basic tastes (Spence, 2019a). At the same time, however, it is important to note that there are several unresolved questions in terms of the cognitive/perceptual underpinnings of such colour–taste crossmodal correspondences. Currently, for example, it is unclear whether colour–taste correspondences are based exclusively on semantic knowledge of particular source objects. Furthermore, it is also unclear to what extent the colour–taste mapping might be mediated by flavour (or rather a specific food/beverage/ingredient), or whether instead it might be one of the correspondences that are mediated by hedonic/emotional associations (Spence, 2020a). As mentioned earlier, it should be born in mind that the weighting of different explanations for colour–taste correspondences might differ depending on the particular basic tastant under consideration. What is more, as yet, there is little data concerning colour correspondences beyond the four or five most commonly mentioned basic tastes. One of the important points to stress here in closing is how very little of the research that has been conducted to date has examined *why* exactly such consistent colour–taste mappings are observed in non-synaesthetic populations.

A number of the methodological limitations with the research that has been conducted to date have also been highlighted. These include the potential role of memory biases as far as the role of an identifiable source object underpinning an individual’s colour–taste mapping is concerned. At the same time, however, when the range of colour options that participants have to match to the basic tastes are restricted, this can lead to possibly suboptimal colour matches—such as, for example, when pink is not provided as a response alternative among those wanting to pick a colour for sweetness (see Spence et al., 2015).

One of the interesting points to highlight is how the most common, or natural, correspondence for the basic tastes would appear to be in terms of colours (hue categories) rather than any other feature, such as, for example, musical (notes or timbres/instrument sounds), textures, or shapes (see Wan et al., 2014). While it might be assumed that this is the most natural mapping because it is the most meaningful, in terms of the visual attribute that is most highly correlated with (and hence the best predictor of) the dominant taste of a food.^21^ However, the fact that olfactory stimuli (in particular, fragrances, i.e., rather than necessarily food aromas) are more frequently paired with, or compared to, musical notes (Deroy et al., 2013; Piesse, 1862/1891) gives rise to the alternative suggestion that is may be the similarity in the way in which stimuli in each modality are structured that determines the mapping that people find most “natural” or intuitive. It may be that the appropriateness of crossmodally matching one pair of senses, or features, rather than another is dependent on the structure of the sensory category (e.g., metathetic vs. prothetic, categorical or not) or on the number of discrete categories, as would appear to be the behind some attempts to match musical notes to hues (see Spence, 2020a).

While, to date, colour–taste associations have been determined either by quizzing people (the participants in psychology experiments, or through survey/questionnaire data) or else on the intuitions of designers (e.g., Favre & November, 1979), one might wonder if, in the not too distant future, it might be possible to establish the existence of crossmodal correspondences based on calculating the semantic distance between relevant terms from online text sources (cf. Caliskan et al., 2017). Alternatively, however, it might also be possible to analyse taste descriptors tagged to colourful (food) images. Are predominantly green (food) images associated with the taste descriptor “bitter” more than with another taste one might ask? However, it is important to note that one potential problem for establishing colour–taste correspondences from assessing semantic distance relates to the fact that colour terms are sometime used inappropriately (e.g., as when we call wine “white,” or steak “bleu/blue”; see also Spence, 2021a, on this theme). Taste words are also sometimes used in a non-gustatory context, such as when the Italian word for sweet “Dolce” is used on a musical score (cf. Jackson et al., in press). Notice also that it may turn out to be easier to predict energy-density/fat-content, or ripeness, from the visual statistics of food images rather than taste/flavour (see Foroni et al., 2016; Motoki, Saito et al., 2021).

Of late, there has been something of an explosion of synaesthetic marketing that uses the colour–taste/aroma/flavour correspondences (e.g., see Spence, 2020c, 2020f). Collaborations between perceptual scientists and designers, marketers, chefs, and culinary artists are providing rich, innovative opportunities to probe and play with these crossmodal correspondences in the context of so-called synaesthetic design (Haverkamp, 2014; Merter, 2017; Spence & Youssef, 2019; Terri Timely, 2009; Velasco et al., 2019; though see also Day, 2011) as well as the emerging field of multisensory experiential marketing (e.g., see Spence, 2012, 2019b; Spence et al., 2014).

## Notes

1 In a fictional story entitled “An Abstract Art,” Jorge Luis Borges and Adolfo Bioy-Casares (1993, p. 72) describe a Parisian restaurant *Les Cinq Saveurs* supposedly opened by Ishmael Querido at the turn of the 19th century. The authors write that: The druggist Payot … began furnishing Querido each week with twelve hundred identical pyramids, each an inch high and each affording the palate one of the now celebrated five tastes—sour, insipid, salty, sweet, and bitter. A veteran of these early campaigns had assured us that at first the pyramids were greyish and translucent and that later on, to make things easier, they were endowed with the five well-known colors, white, black, yellow, red, and blue. Unfortunately, however, the crossmodal mapping between tastes and colours in this literary example is never revealed.

2 Notice here how the suggestion that the taste affected the patient in the same way as the colour, makes it sound rather like emotional mediation, or matching (see Emotional mediation of crossmodal correspondences section).

3 According to Joseph Boehme (1912):
There are commonly four colours, as white, yellow, red, and green: Now according to what colour the fruit (as an apple) is most signed, accordingly is the taste also in the salt; as white with a clear thin skin somewhat inclining to dark gives sweetness, which is of Venus property; if the sweet taste be strong and powerful, then Jupiter is potent therein; but if it be weak and fulsom, then the moon is strong therein; but if it be hard, and of a brownish colour, then Mars is strong therein; but if the white colour be of a grayish-brown, then Saturn is strong therein: Venus makes a white colour, Mars red, and bitter in the taste; Mars makes Venus’s colour light, Mercury gives a mixt colour, and opens the green in Mars; Jupiter inclines to blue, Saturn to black, almost gray; the sun makes the yellow colour, and gives the right sweetness in the salt, and casts forth the pleasant smell, which takes its rise from Sulphur; Saturn makes astringent [sharp] and sour; and each property represents itself externally, as it is internally in the dominion, so also by the form [or signature] of the leaf, or branches. (pp. 85–86)

4 A styptic substance is one that is capable of causing bleeding to stop when it is applied to a wound.

5 Here, it is worth noting that while coloured coffee cups have recently been shown to prime the associated taste qualities (e.g., pink-sweet; green and yellow-sour), there is less evidence that cups with metallic appearance set metallic taste expectations (see Carvalho & Spence, 2019, 2021).

6 Piesse explicitly notes how sounds and odours blend together similarly, producing different degrees of “a nearly similar impression” in the sensory nerves (Piesse, 1867, p. 39). For example, the mixture needed to prepare the odours for the handkerchief evokes effects on the smelling nerve “similar to that which music or the mixture of harmonious sounds produces upon the nerve of hearing, that of pleasure” (Piesse, 1867, p. 219). (Again, note the appeal to affective correspondences.) Remarkably, Piesse states that creating a mixture of scents is like creating a mixture of sounds, that is, chords: “We have citron, lemon, orange peel, and verbena, forming a higher octave of smells, which blend in a similar manner” (Piesse, 1867, p. 39). The pleasantness of musical harmony resembles that of perfumes, and Piesse presents a scale of correspondences between sounds and odours (this, the so-called scent scale). Piesse believed that “there is, as it were, an octave of odours like an octave in music” (Piesse, 1867, p. 38).

7 Writing a little more than 150 years ago, C. S. Peirce (1868) puts it thus: “Sight by itself informs us only of colors and forms [and that] No one can pretend that the images of sight are determinate in reference to taste.”

8 The one special colour–taste correspondence might be in terms of redness and sweetness, given the suggestion that colour vision may have evolved in primates to help discriminate this colour from among the green canopy (e.g., Sumner & Mollon, 2000, 2003). It is also worth noting, in passing, that more of the visual cortex given over to processing pinkish-red skin tones (Changizi et al., 2006) and that, across cultures, if a language contains only three colour terms, then these terms are always white/light, black/dark, and red (Berlin & Kay, 1969), again hinting that not all colours are treated equally.

9 Prior to the advent of synthetic chemistry, colouring foods would typically been achieved by means of the use of herbs and spices (see Woolgar, 2018). Note here only how saffron (which gives food a golden appearance) has at various points in history been the world’s most expensive spice, by weight (Spence, in press).

10 Note that Kandinsky (1977) also raised the possibility of mediated colour–taste correspondences when he suggested that “One might say that keen yellow looks sour, because it recalls the taste of a lemon” (p. 24).

11 Furthermore, other basic tastants, such as caffeine (bitter), citric acid (sour), and monosodium glutamate (umami), when presented in pure crystalline form are all whitish in appearance too. However, most people are much less familiar with them as they are rarely encountered outside the confines of the food science laboratory (cf. Wang et al., 2016). All this to say that it would seem unlikely that pure tastants necessarily constitute the source object underpinning colour–taste crossmodal correspondences.

12 While sugar has been refined for more than 2,500 years (Horton et al., 2015), in Western Europe, it was a rare delicacy, traditionally considered a spice, given its exoticism, rarity, and price (Mintz, 1985). It was only when prices started to fall, for example, in the UK in the 1700s, as a result of the slave trade in the Indies that the majority of people would likely have started to become familiar, at least first hand, with sweetness in the form of crystalline white sugar. As such, it would seem that the crossmodal colour–taste correspondence between white and sweet mentioned by Hieronymus Cardanus, when he linked Venus to whiteness and sweetness in the 16th century (see Classen, 1998, p. 167), must have been based on something other than the refined white sugar that so many of us are familiar with today.

13 The crossmodal correspondence between the trigeminal burn of chile and the colour red (e.g., Shermer & Levitan, 2014; Spence, 2018a), while undoubtedly constituting another example of a colour correspondence with the chemical senses, falls outside the scope of the present review.

14 Indeed, it has previously been suggested that one reason why it may be harder to modify the perception of saltiness using colour than to modify sweetness, say, is that salty foods come in so many different colours (see also Maga, 1974; Spence et al., 2010).

15 Here, it is perhaps also worth noting how the colours that synaesthetes associate with letters has been shown to be predictable, at least in part, based on the colours of the objects that they happen to associate with words that start with the letter (see Mankin & Simner, 2017). So, for example, the letter “A” often makes people think of “Apple,” and apples are typically either green or red, hence explaining why these two responses are particularly common concurrent colours in the case of coloured grapheme/phoneme synaesthesia.

16 Averaging would perhaps make more sense when matching the intensity of oral sensations and the saturation of hues (e.g., Green et al., 1993; Panek & Stevens, 1966).

17 Although note that the suggestion that crossmodal correspondences are underpinned by similarity in arousal (Sievers et al., 2019) would seem more appropriate to explaining audiovisual correspondences that to accounting for colour–taste correspondences.

18 Note also how colours are not only associated with basic tastes but also with a range of other sensory qualities. So, for example, red and blue are associated with hot and cold when thinking about temperature (Spence, 2020e), but with sweet and salty when thinking about basic tastes (Spence, 2019a).

19 You can listen to the Audio UX track Vivid Nostalgia at https://www.youtube.com/watch?v=SVa6eQ1oRt8

20 In 2015, artist Sean Rogg curated an event in which members of the public, who had been asked to dress monochromatically, were “invited to taste colour.” The visitors got to drink fine wine and watch a host of dancers perform. According to the artist, the soundscapes were supposed to sound like their respective colours while also pairing well with the wines (Miller, 2015).

21 As Stummerer and Hablesreiter (2010) puts it, “Colour clearly conveys to the brain what taste is to be expected” (p. 86).
